# *In Vitro* Evaluation of Bacterial Leakage at Implant-Abutment Connection: An 11-Degree Morse Taper Compared to a Butt Joint Connection

**DOI:** 10.1155/2016/8527849

**Published:** 2016-05-03

**Authors:** Hooman Khorshidi, Saeed Raoofi, Afagh Moattari, Atoosa Bagheri, Mohammad Hassan Kalantari

**Affiliations:** ^1^School of Dentistry, Shiraz University of Medical Sciences, Shiraz, Iran; ^2^School of Medicine, Shiraz University of Medical Sciences, Shiraz, Iran

## Abstract

*Background and Aim*. The geometry of implant-abutment interface (IAI) affects the risk of bacterial leakage and invasion into the internal parts of the implant. The aim of this study was to compare the bacterial leakage of an 11-degree Morse taper IAI with that of a butt joint connection.* Materials and Methods*. Two implants systems were tested (*n* = 10 per group): CSM (submerged) and TBR (connect). The deepest inner parts of the implants were inoculated with 2 *μ*L of* Streptococcus mutans* suspension with a concentration of 108 CFU/mL. The abutments were tightened on the implants. The specimens were stored in the incubator at a temperature of 37°C for 14 days and the penetration of the bacterium in the surrounding area was determined by the observation of the solution turbidity and comparison with control specimens. Kaplan-Meier survival curve was traced for the estimation of bacterial leakage and the results between two groups of implants were statistically analyzed by chi-square test.* Results*. No case of the implant system with the internal conical connection design revealed bacterial leakage in 14 days and no turbidity of the solution was reported for it. In the system with butt joint implant-abutment connection, 1 case showed leakage on the third day, 1 case on the eighth day, and 5 cases on the 13th day. In total, 7 (70%) cases showed bacterial leakage in this system. Significant differences were found between the two groups of implants based on the incidence of bacterial leakage (*p* < 0.05).* Conclusion*. The 11-degree Morse taper demonstrated better resistance to microbial leakage than butt joint connection.

## 1. Introduction

**Figure 1:**
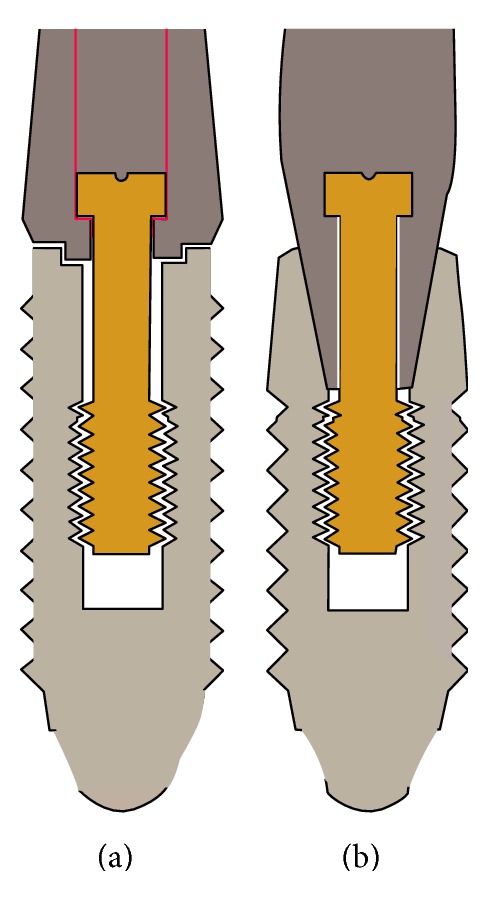
The butt joint connection (a) and the internal Morse connection (b).

Dental implant is one of the common methods for replacing missing teeth with a high success and excellent survival rate [1]. Most common implant systems consist of two main parts: an endosteal fixture and a prosthesis-supporting abutment connected to fixture with a screw. Fixture-Abutment Interface (FAI) connection can be external or internal. The internal connection implants are currently more common than the external type. Bacterial colonization of abutment implant interface in two part implant systems is a major challenge in implant dentistry [2]. It has been proposed that the abutment implant connection gap inside the implant cavity could act as reservoirs for pathogens that cause biological problems leading to peri-implantitis [3]. In fact, the bacterial leakage in the implant-abutment interface (IAI) is raised as the most important factor in the occurrence of inflammatory reactions around the implant. Regardless of stability, design and engineering of abutment implant connection may have a greater impact on bacterial seal. Implants should, therefore, be fabricated with perfect seal to prevent or limit any biofilm accumulation. It has been stated that a good marginal fit of implant components seemed to be able to prevent bacterial leakage [4]. The available implant systems in the market utilize different IAI. Regarding sealing ability, in our opinion, two main IAIs are the internal Morse connection and the butt joint connection (Figure 1). The Morse taper connection has an angle of 6 to 16° between implant and abutment. Some studies utilizing different tapering systems have described the presence of bacterial leakage at the IAI. So, the aim of this study was to compare the bacterial leakage of an 11-degree Morse implant-abutment connection with a butt joint connection.

## 2. Materials and Methods

Ten implants of each connection type, 11° internal Morse connection (CSM Implant, Daegu, Korea) with a diameter of 4.2 mm and a length of 12 mm, and butt joint connection (TBR, TBR Implant Groups, Toulouse, France) with a diameter of 4 mm and a length of 13 mm were utilized for the evaluation of microleakage.

### 2.1. The Preparation of Standard Strain Bacteria

The bacteria were purchased and ordered from Iran Industrial Scientific Research Organization with PTCC = 1683 specifications. Therefore, they were incubated in TBS (Tryptic Soy Broth) and recovered. Afterwards, the bacteria with Glycerol 15% in the storage form were put in the freezer in 70°C to be used in the future stages of the study.

### 2.2. Preparation of* Streptococcus mutans* Suspension with McFarland 0.5 Turbidity

To do this, the saved bacteria were transmitted about 20 microliters in blood agar environment with 5% sheep blood and incubated linearly. The bacteria were incubated in the 37°C incubator in microaerophile condition (CO_2_ 5%) for about 24–48 hours. Then, some similar mono colonies were incubated in 2 mm TSB environment and reached 0.5% McFarland turbidity (equals 108 CFU/mL).

By the application of micropipette automatic control, 2 microliters of* Streptococcus mutans* suspension with 108 CFC/mL concentrations was taken and put under the sterilized condition in the deepest part of inner part of implants. Then, the related abutment was tightened on the implants with 30 N·cm standard torque under sterile conditions.

As positive control group samples, two similar laboratory tubes were prepared which contain (TBS) nutritious solution and mixed with 2 microliters of* Streptococcus mutans* suspension which shows the existence and growth of bacteria through solution turbidity and bacteria incubation and confirmed the microorganism permanence in the whole experiment.

As negative control group samples, two similar tubes were used as well which were filled with brain-heart infusion sterile nutritious solution and were confirmed with solution transparency and usual incubation technique.

To estimate the unwanted pollution of outer surface of implant, the following pollution and implant blocking, each implant-abutment complex was plunged into the separate sterile nutritious solution for about 1 minute and moved with rolling movements (total of 20 experimental tubes). The tubes containing turbid solution which were the signs of outer surface pollution were excluded from the study. Each implant-abutment set was put in Eppendorf sterile tubes containing 150–200 cc nutritious solution in a way that solution level is about the connection level of implant-abutment.

In the next step, 20 tubes containing samples, 20 test tubes pollution control tubes of outer surface, 2 (+) control samples, and 2 (−) control samples were put inside the incubator with 37°C for about 14 days. The samples were incubated and tested bacterially daily, the penetration of bacteria to the surrounding environment through solution turbidity and its comparison with negative control sample and colonization on blood agar environment is determined, and finally, the existence or nonexistence of turbidity and bacteria colonization in considered date was recorded.

### 2.3. Colony Calculation

To calculate the colony, 5 microns of turbid tubes was taken at the appointed time and incubated in sheep blood agar plate and scattered by an L bar completely. The plates were kept in the incubator for 24–48 hours and the number of produced colonies was calculated and eventually the number of bacteria was estimated with the following formulae: the number of calculated colonies × thinness quotient × volume (mL).

The frequency (number and percentage) of bacteria leakage in different days in each of the two implant systems was determined and reported. The Kaplan-Meier survival curve for the incidence of leakage in different days in TBR group was drawn and the groups' difference of implant-abutment connection considering the amount of leakage incidence with chi-square test was statistically analyzed.

## 3. Results

The present study was carried out for 14 days and the number of samples in each group was 10. There was no case with bacterial leakage and no turbidity of the solution was observed for the 11-degree Morse taper group (Figure 3).

**Figure 2:**
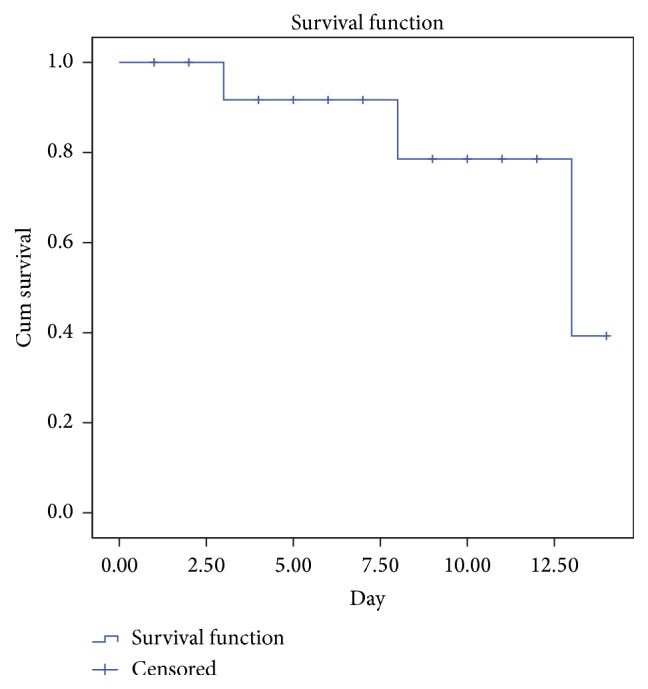
Kaplan-Meier survival curve.

**Figure 3:**
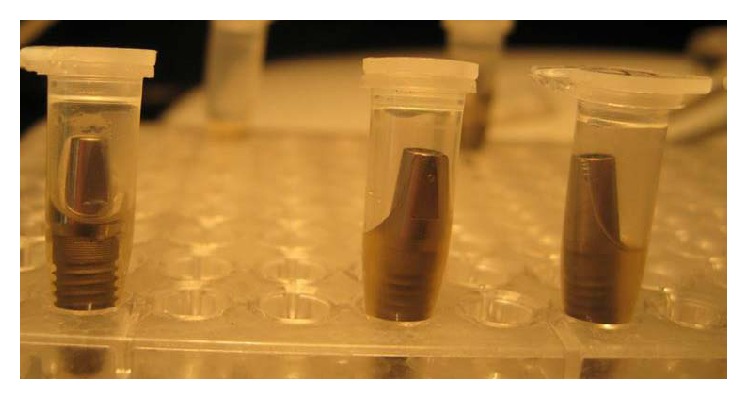
The samples from butt joint connection group with (two samples on the right side) and without turbidity.

In butt joint connection samples, the signs of leakage were observed in 7 out of 10, one on the 3rd day, one on the 8th day, and 5 on the 13th day. In all samples, some colonies were separated two days before turbidity observance with incubation. The Kaplan-Meier survival curve in the investigation of leakage incidence during the 14-day period is illustrated in Figure 2 and the result based on the number of CFU is presented in Table 1. The difference was statistically significant based on chi-square test (*p* < 0.05).

## 4. Discussion

During the 14-day* in vitro* evaluation of the bacterial leakage at implant-abutment interface (IAI), in butt joint connection samples, the signs of leakage were observed in 70% of cases. The 11-degree Morse taper demonstrated no sign of microbial leakage, thus, better resistance to microbial leakage than a butt joint connection design. Bacterial seal of IAI is of the utmost importance in the establishment of stable hard and soft tissue integration [2]. In butt joint connection, surfaces form a 90-degree angle; but in conical connection, there is a connection between the two funnel surfaces and one of them goes down to the other. The Morse taper connection seems to be more efficient based on these biological aspects [5]. The inner part of the two piece implants has the potential to act as a reservoir of bacteria and their by-products [6]. So, implants should be fabricated with perfect seal to prevent or limit any inflammatory reactions leading to bone loss around implants. Precision in the manufacturing process and engineering parts is of particular importance. The connection type design is more important. The Morse taper connection has been indicated for single implants, fixed partial prostheses, and overdenture planning, since it exhibits high mechanical stability [7]. The design and characteristics of IAI are proposed to mainly achieve the reduced crestal bone stress. Emphasis on stress distribution should not ignore the importance of IAI leakage ability. Hence, the precision in the manufacturing process and engineering parts is of particular importance. The connection type design is more important.

No case of bacterial leakage and turbidity of the solution were observed for the 11° Morse taper group. Lower bacterial leakage of Morse taper connection was reported in other studies and was also confirmed in the present study as there were no cases of 11° Morse taper connection showing microbial leakage during the 14-day period of evaluation. Tripodi et al. [8] evaluated the bacterial leakage of cone Morse taper IAI with and without loading and did not find any differences in microbial leakage between two groups. They utilized the identical implants in their study. D'Ercole et al. [9] observed the leakage over a period of 28 days in cone Morse taper internal connections and in screwed-abutments connections and reported the lower infiltration rates of cone Morse taper internal connections. Aloise et al. [10] compared the frequency of bacterial leakage along the IAI between two systems of Morse taper dental implants and found no statistical differences between them. Among the studies about leakage on Morse taper connection, only few were related to 11° Morse taper connection. To the best of our knowledge, this is the first study that compared the microbial leakage of 11° Morse taper connection with that of a butt joint interface.

Berberi et al. [11] studied the three brands of implant systems with the same 11° conical connection and concluded that the tested connections appear to be unable to prevent leakage. Although the accuracy in fabrication and the precision of fit of the components seem to be an important factor in resistance to leakage, nevertheless, we utilized a CE approved 11° Morse taper connection randomly from available brands in the market.

Teixeira et al. [3] observed higher degrees of bacterial leakage in Morse taper connections (77%) and internal hexagon (100%) after seven days. Tesmer et al. [12] compared implants with internal conical implant-abutment connections and screwed trilobed connections. They proved that only 30% of conical connection implants had bacterial leakage. Merz et al. [13] compared the 8-degree Morse taper and the butt joint connections and concluded that the mechanics of conical abutment in a three-dimensional, nonlinear finite element model was superior to that of the butt joint connections. On the other hand, Shin et al. [14] revealed that the external butt joint was more advantageous than the internal cone in terms of the postload removal torque loss. In their study, in the two-stage internal cone system, the wide-diameter group demonstrated a significantly lower loss rate than the regular-diameter group. This torque loss can affect the resistance of IAI to bacterial leakage. Steinebrunner et al. [15] have utilized loading process in the evaluation of bacterial leakage and showed significant differences between the implants and the number of loading cycles. Our investigation was in static condition, without loading; therefore, torque loss was not an issue related to our results.

It is believed that a good marginal fit of implant components seemed to be able to prevent bacterial leakage [16]. Nevertheless, there is a lack of documentation about the impact of the implant-abutment connection on the crestal bone level changes, although the positioning of the machined neck and microgap may limit crestal bone level changes at nonsubmerged implants [17]. The design and engineering of IAI may have a greater impact on bacterial seal. Schmitt et al. [5] in a recent systematic review indicated that implant systems utilizing a conical IAI provide better results in terms of abutment fit, stability, and seal performance. Tripodi et al. [8] evaluated the bacterial leakage of cone Morse taper IAI with and without loading and could not find differences in microbial leakage between two groups. They utilized the identical implants in their study. In another study, Tripodi et al. [18] reported no significant differences in leakage observed in internal hexagon and Morse taper IAIs. They utilized a cone taper implant and found bacterial leakage in 2 of 10 implant-abutment connections. In our study, we could not find any bacterial leakage during the 14-day period in the 11° Morse taper group. Difference in the results may be due to difference in the design of implants and degrees of tapering in implant systems. Although it is reported that implant systems using a conical IAI provide better results in terms of seal performance, in most studies, some leakage in different days has been reported. Using stimulator, Koutouzis et al. [19] reported bacterial leakage in only one of fourteen implants with internal conical connections. In another study by Aloise et al. [10], bacterial leakage was observed in 2 of 10 implants with internal conical connection. Berberi et al. [11] used internal connection implants in their study and bacterial leakage was confirmed in them. Dibart et al. [20], who reported no leakage, assessed the presence of bacterial leakage for only 72 h. An explanation for this diversity in results may be due to differences in Morse design and tapering degrees in the investigated implant samples. Among other factors affecting bacterial leakage are closing torque rate during the tightening of abutment on the fixtures. We used a 30 N·cm torque for all implants in both groups. In general, it seems that Morse taper connection has an obvious advantage regarding sealing ability; nevertheless, other aspects regarding stability, stress distribution, screw loosening, and platform switching must be considered in IAI design and engineering.

## Figures and Tables

**Table 1:** Assessment of bacterial growth in butt joint connection group.

Sets (butt joint)	CFU
1	8
2	0
3	7
4	12
5	16
6	12
7	13
8	10
9	0
10	0
Mean	11.14
Standard deviation	9.5
